# Broad Perfect Transparent Band in Asymmetric Photonic Crystals with Graded-Index Films

**DOI:** 10.3390/mi17080976

**Published:** 2026-08-19

**Authors:** Chenming Zhang, Qi Wei, Xinya Zhang, Wufeng Sun, Xiangyu Li, Ang Liu, Guiqiang Du

**Affiliations:** School of Space Science and Technology, Shandong University, Weihai 264209, China

**Keywords:** photonic crystals, graded-index films, transfer matrix method, perfect transparent band, broadband transmission

## Abstract

Photonic crystals possessing mirror symmetry have been widely investigated to obtain perfect transmission properties. However, complex asymmetric nanostructures can achieve perfect transmission via phase-matching, while one-dimensional photonic crystals exhibit pronounced angle- and polarization-dependent transmission characteristics under oblique incidence. In this study, we systematically investigated the optical properties of one-dimensional asymmetric photonic crystals containing graded-index films, which yielded a broad perfect transparent photonic band that differed from those occurring in one-dimensional asymmetric photonic crystals comprising solid-index films. The transparent band redshifted, and its bandwidth decreased with increasing amplitude of the graded index. Moreover, the broad perfect transparent band obtained over a wide range of incident angles exhibited greater insensitivity to the incident angle for transverse magnetic polarization than for transverse electric polarization. These results provide a theoretical foundation for designing perfect broadband optical devices using photonic crystals with graded-index films.

## 1. Introduction

Photonic crystals are nanostructures with periodically modulated dielectric constants, which form photonic bandgaps that prohibit optical propagation in photonic crystals [1,2,3]. This property makes photonic crystals widely applicable to optical controlling, sensing, and radiative thermal management [4,5,6,7,8,9]. In conventional multilayers and homogeneous dielectric systems, an abrupt change in refractive index typically produces significant Fresnel reflections, whereas graded-index designs effectively reduce interfacial reflections, enhance transmission, and improve impedance matching [10,11,12]. Because metallic materials suffer from optical losses, achieving perfect transmission using metal-containing nanostructures is difficult [13]. In contrast, all-dielectric photonic crystals are generally used to realize perfect broad wide-angle transmission bands [14].

Photonic crystals possessing mirror symmetry have been widely explored to achieve perfect transmission properties. Early studies demonstrated that one-dimensional metal-dielectric periodic structures can form tunable transparent bands through interference effects [15] where the loss in the metals was not considered. Studies of the physical mechanism of the transparent photonic band indicated that symmetry played an important role in the formation and classification of perfect transmission [15,16,17]. However, nanostructures with perfect symmetry are difficult to fabricate experimentally. Moreover, complex asymmetric nanostructures achieved perfect transmission under appropriate phase-matching conditions [18,19,20,21]. Nevertheless, prior studies have predominantly focused on photonic bandgaps and the evolutionary features of defect modes in symmetric photonic systems. Fewer studies focused on the perfect transmission passbands arising from asymmetric configurations. The comprehensive principles of transparent photonic bands in one-dimensional asymmetric photonic crystals containing graded-index thin films still remain poorly investigated.

Moreover, one-dimensional photonic crystals exhibit pronounced angle- and polarization-dependent transmission characteristics under oblique incidence, which facilitates further comparison of the transparent bands under transverse magnetic (TM) and transverse electric (TE) polarizations [22]. Previous studies on the transmission properties of one-dimensional photonic crystals containing gradient materials or graded defect layers have revealed that graded layers significantly affect the position and bandwidth of photonic bandgaps and defect modes [23,24]. By varying the refractive-index profile, the interfacial optical impedance in graded-index films can be effectively tailored [25,26].

In the present work, we achieve a wide perfect transmission photonic band within one-dimensional asymmetric photonic crystals integrated with graded-index thin films. This passband differs distinctly from that observed in asymmetric photonic crystals constructed using constant-index dielectric layers. Furthermore, we systematically analyze how the graded-index profile, incident angle, and polarization state modulate the transmission band.

The proposed nanostructures possess important application prospects for transmissive optical lenses. Existing plasmonic environmental sensors are constrained by Ohmic losses [27]; conversely, the broadband perfect transparent bands in all-dielectric photonic crystals circumvent these limitations for wide-angle, low-loss optical sensing applications. Moreover, they can serve as broadband anti-reflection filter coatings for wide-angle imaging lenses, wide-angle vehicle optical sensing lenses, and visible-to-near-infrared transmission lenses under multi-incident-angle working conditions [28]. Conventional uniform thin-film lenses suffer from severe transmittance attenuation and spectral distortion at large incident angles; by contrast, the asymmetric photonic crystals containing graded-index layers effectively suppresses multiple reflections in the passband and maintain perfect transmittance over a broadband and wide incident angle range.

## 2. Theoretical Fundamentals of Asymmetric Photonic Crystals and Graded-Index Thin Films

The transfer matrix method [29] is first adopted to calculate the optical properties of photonic crystals containing solid-index films, where a one-dimensional photonic crystal is constructed by alternately depositing thin films with two different refractive indices ($n_{a}$ and $n_{b}$) and distinct thicknesses. Within the framework of the transfer matrix method, the interaction between incident light waves and thin films can be described by the characteristic matrix M in the one-dimensional photonic crystal. Let the field vectors on two sides of each thin film be *E*_1_, *H*_1_, *E*_2_ and *H*_2_, respectively; their correlation can be expressed as

(1) $$\left[\begin{array}{l} E_{1}\\ H_{1} \end{array}\right]=M\left[\begin{array}{l} E_{2}\\ H_{2} \end{array}\right]$$

The phase thickness in an individual dielectric layer is denoted by *δa*, which depends on the refractive index of the dielectric layer during the propagation of the electric vector from interface 1 to interface 2. The transfer matrix M in a layer a can be written as

(2) $$M=\left[\begin{array}{cc} \cos\delta_{a} & -\frac{i}{\eta_{a}}\sin\delta_{a}\\ -i\eta_{a}\sin\delta_{a} & \cos\delta_{a} \end{array}\right]$$

In the formula, the effective optical admittance $\eta_{a}$ is related to different polarization with

(3) $$\eta_{a}=\left\{\begin{array}{l} n_{a}/\cos\theta \;\;(TM)\\ n_{a}\cos\theta \;\;\;\;(TE)\; \end{array}\right.$$

For a one-dimensional photonic crystal composed of multiple layers, the electric and magnetic field vectors at the interfaces of each film can be solved layer by layer. For an *N*-layer dielectric stack, recursive calculations are carried out layer by layer to obtain the relationship between the electromagnetic fields at the incident surface and exit surface of the photonic crystal, which is expressed as

(4) $$\left[\begin{array}{l} E_{1}\\ H_{1} \end{array}\right]=\left(M_{1}M_{2}\ldots M_{N}\right)\left[\begin{array}{l} E_{N+1}\\ H_{N+1} \end{array}\right]$$

in which the final characteristic matrix in the one-dimensional photonic crystal is obtained via the above recursion.

**Figure 1:**
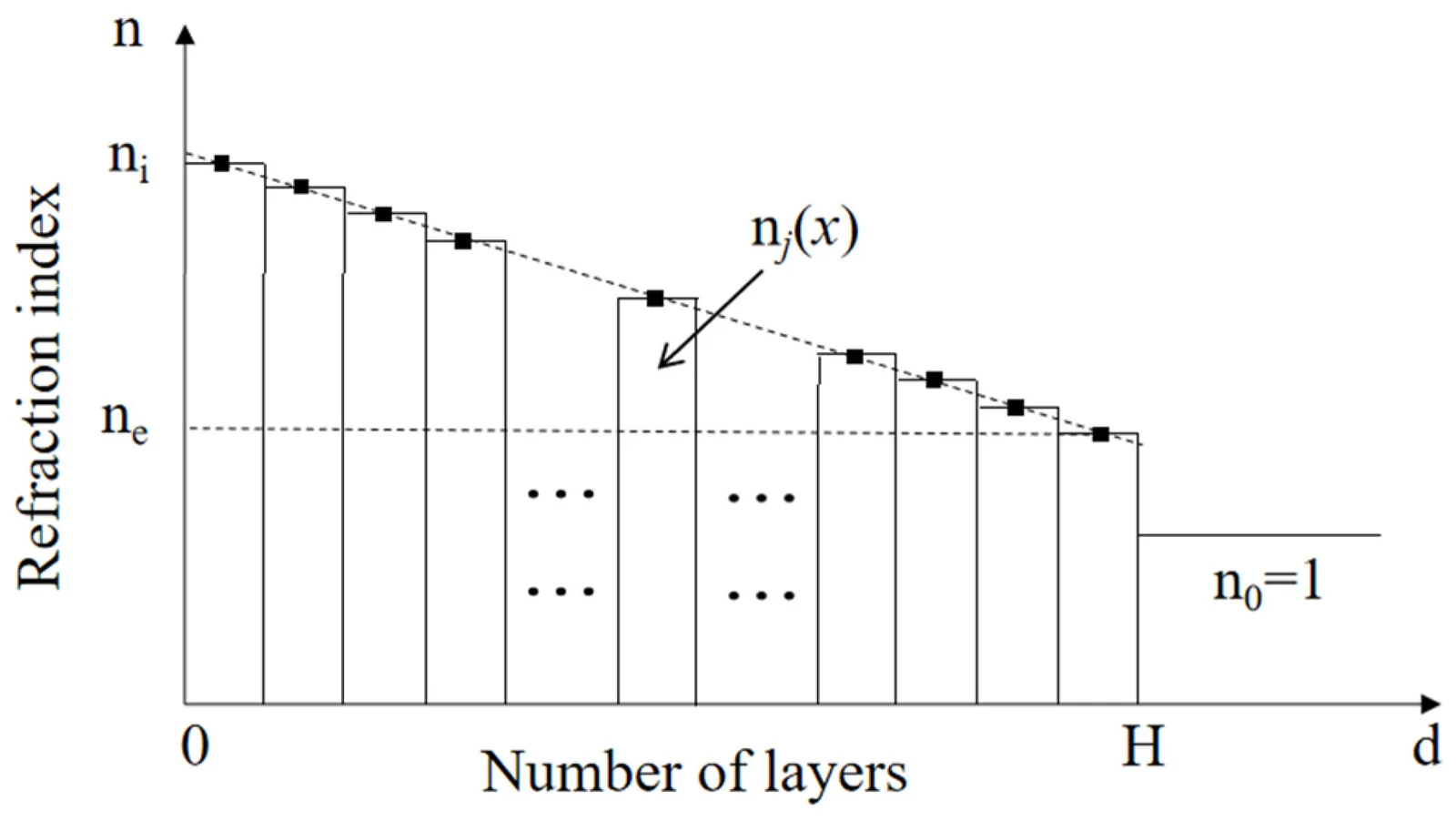
Schematic of the layered structure in a linearly graded-index thin film.

For graded-index thin films, the differential transfer matrix method [30] can be used generally to simulate their optical properties. In this paper, linearly graded-index thin films are considered in the photonic crystals, where their transmission and reflection can be simulated approximatively using the layered medium theory [31]. The linearly graded-index thin film is divided into *N* sublayers with same thickness h, where every sublayer has a fixed refractive index $n_{j}(x)$, as shown in Figure 1, where the refractive index of the *j*-th sublayer is then taken as

(5) $$n_{j}(x)=\frac{n_{j}((x-1)h)+n_{j}(xh)}{2}$$

And there is *j* = 1, 2, …, *N*. The refractive index of the graded-index thin film varies from $n_{i}$ to $n_{e}$ linearly. Let the refractive indices of air be *n*_0_. The thickness of every sublayer is expressed by

(6) $$h=\frac{H}{N}$$

where H is the total thickness of the linearly graded-index thin film.

For an incident monochromatic wave of wavelength *λ* at an angle *θ*_0_, the refraction angle *θ_j_* in the *j*-th sublayer is determined by Snell’s law. The phase thickness *δ_j_* of the *j*-th sublayer is given by:

(7) $$\delta_{j}=\frac{2\pi }{\lambda }n_{j}h\cos\theta_{j}$$

The characteristic matrix $M_{j}$ in the *j*-th sublayer can be written as

(8) $$M_{j}=\left[\begin{array}{cc} \cos\delta_{j} & -\frac{i}{\eta_{j}}\sin\delta_{j}\\ -i\eta_{j}\sin\delta_{j} & \cos\delta_{j} \end{array}\right]$$

where the effective optical admittance $\eta_{j}$ of each sublayer is determined by the polarization. For TE polarization and TM polarization, the optical admittance is expressed as:

(9) $$\eta_{j}=\left\{\begin{array}{l} n_{j}/\cos\theta_{j}\;\;\;\;(TM)\\ n_{j}\cos\theta_{j}\;\;\;\;\;\;\;(TE) \end{array}\right.$$

By sequentially multiplying the characteristic matrices of all *N* sublayers, the total characteristic matrix of the graded-index film is obtained, so that the reflection coefficients, transmission coefficients, and other optical properties can be derived.

## 3. Numerical Simulation and Analysis on Optical Properties of Photonic Crystals

**Figure 2:**
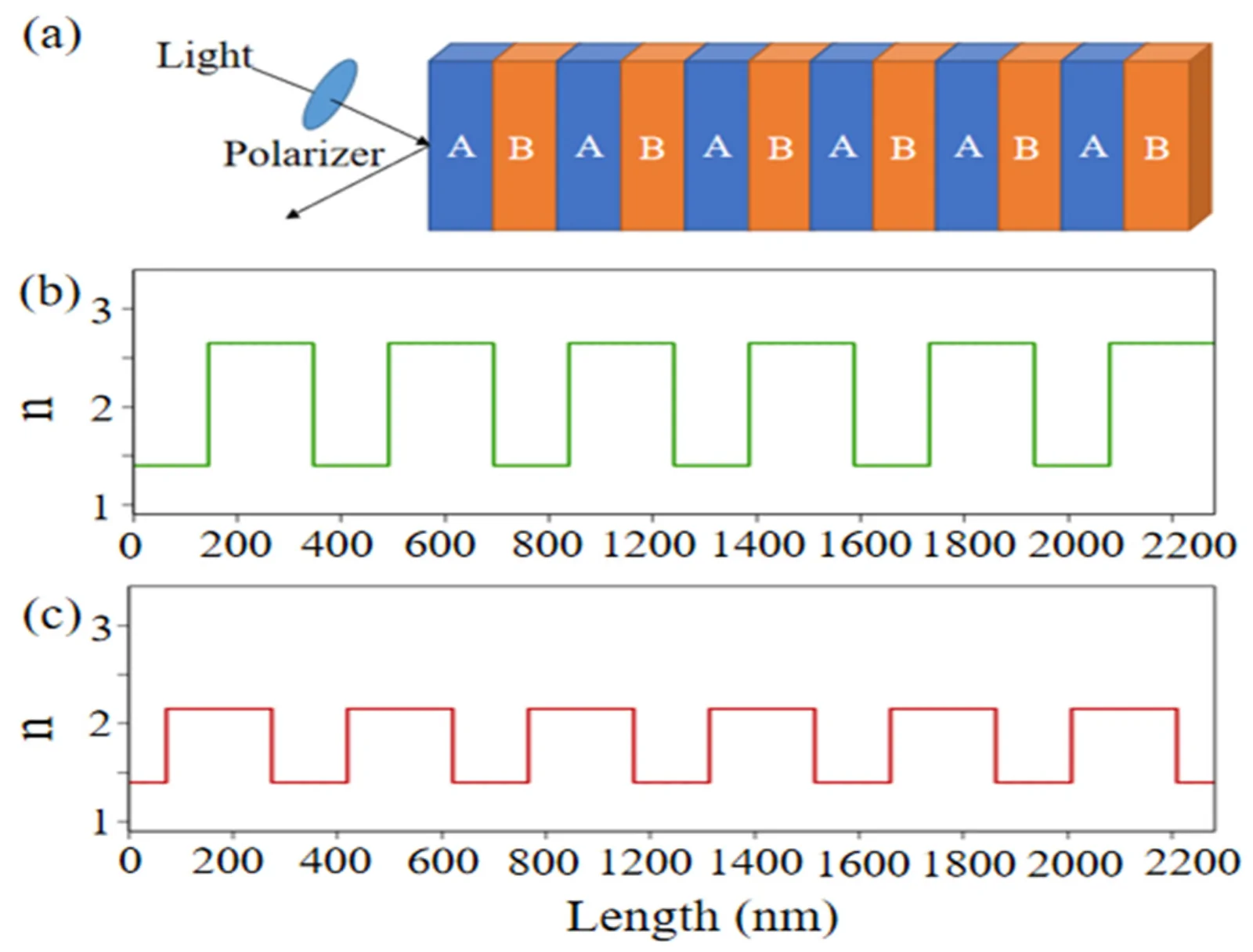
(**a**) Schematic of the photonic crystal (AB)*^N^*, where A and B represent different materials with d_A_ = 145 nm and d_B_ = 202 nm, and *N* is period number. (**b**) Refractive-index profile in the asymmetric photonic crystal (AB)^6^ with solid-index films. (**c**) Refractive-index profile of the symmetric photonic crystal (A_1/2_BA_1/2_)^6^ with solid-index films.

First, we numerically simulated the optical properties of two different one-dimensional photonic crystals with solid-index films using the transfer matrix method and investigated the differences between their transmission bands. Figure 2a shows the schematic of an asymmetric photonic crystal (AB)*^N^*, where A and B are different materials, and *N* is the periodic number. Figure 2b,c present the refractive-index profiles as a function of the length for the symmetric structure (A_1/2_BA_1/2_)*^N^* and for the constant-index asymmetric photonic crystal (AB)*^N^*, respectively, where both comprise solid-index films.

**Figure 3:**
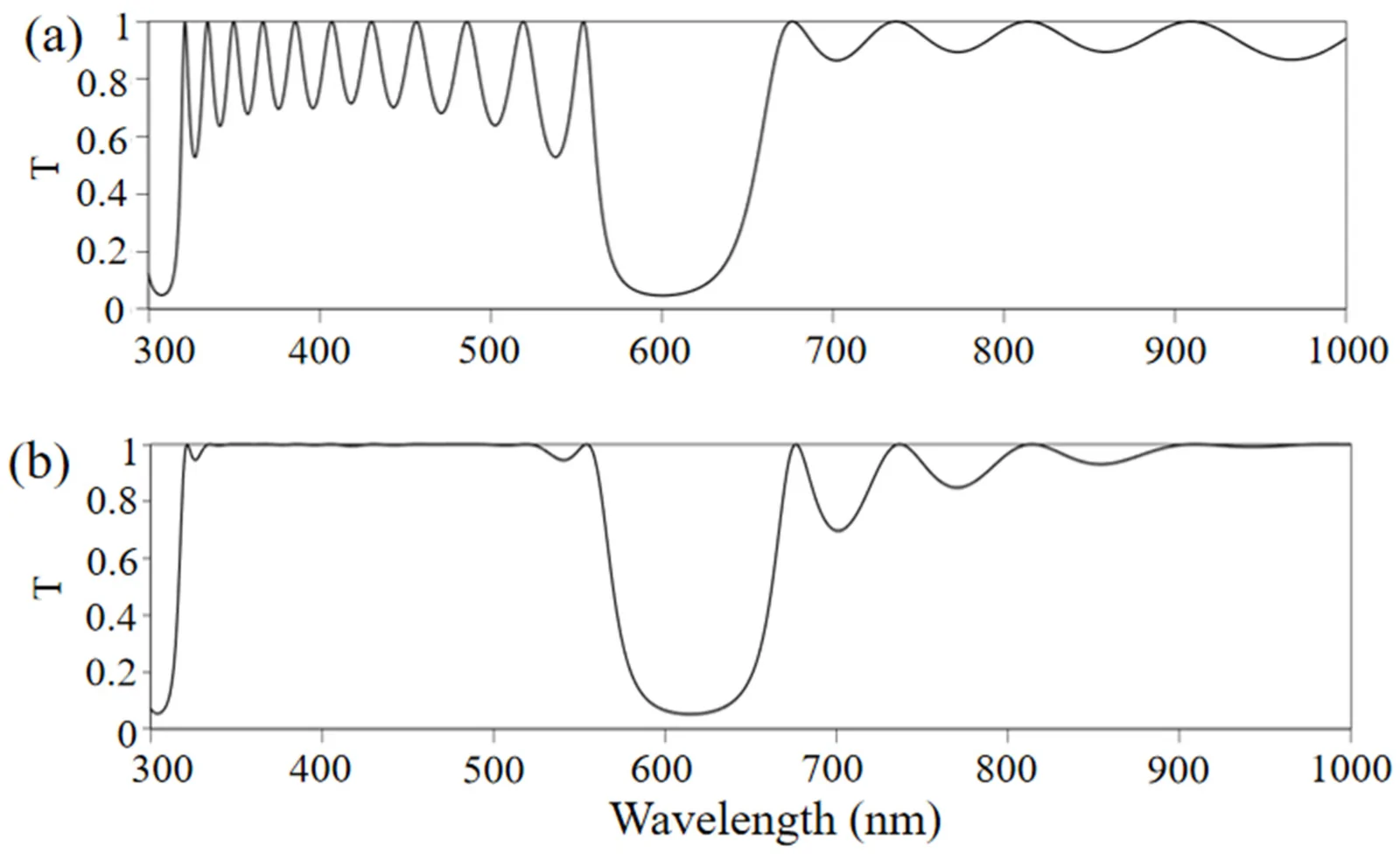
Transmission spectrum of different photonic crystals with solid-index films at normal incidence for the (**a**) asymmetric structure (AB)^6^ and (**b**) symmetric structure (A_1/2_BA_1/2_)^6^.

For the two photonic crystals composed of solid-index films, (AB)*^N^* and (A_1/2_BA_1/2_)*^N^*, (Figure 2b and Figure 2c, respectively), the refractive indices of layers A and B were *n*_A_ = 1.4 and *n*_B_ = 2.1, respectively, and the period number was *N* = 6. The physical thicknesses of the two materials were d_A_ = 145 nm and d_B_ = 202 nm. First, the transmittances of (AB)^6^ and (A_1/2_BA_1/2_)^6^ as functions of the wavelength and at normal incidence are shown in Figure 3a,b. A partially transparent band was observed in the asymmetric photonic crystal (AB)^6^ (Figure 3a), whereas a broad perfect transparent band was realized in the symmetric photonic crystal (A_1/2_BA_1/2_)^6^ (Figure 3b).

**Figure 4:**
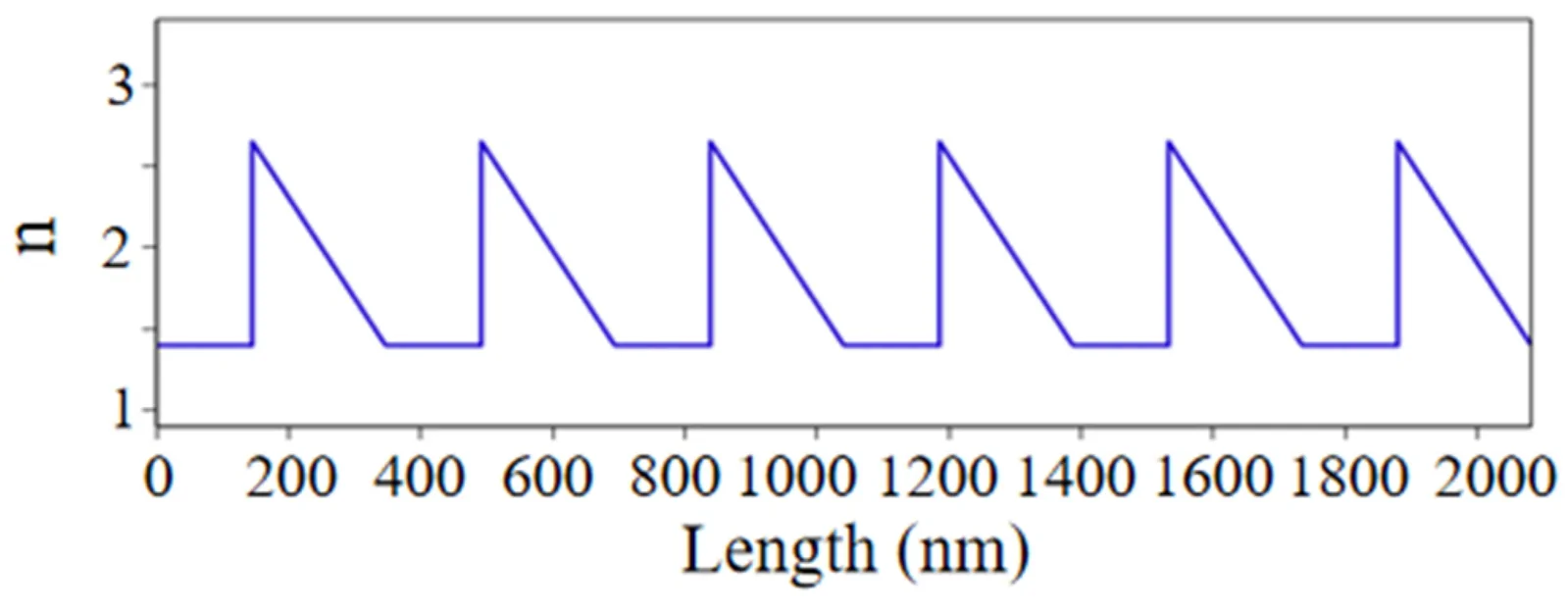
Refractive-index profile in the asymmetric photonic crystal (AC)^6^ with the graded-index film C. Here, d_A_ = 145 nm and d_C_ = 194 nm.

Second, to overcome the restriction of obtaining a wide perfect passband, which generally depends on photonic crystals with mirror symmetry, an asymmetric photonic crystal (AC)^6^ with graded-index films was considered. The refractive-index profile is shown in Figure 4. The layer C in the asymmetric photonic crystal (AC)^6^ comprised a graded-index film whose refractive index decreased linearly from 2.65 to 1.55 within its thickness. The mean refractive index of this graded film was 2.1, which was identical to that of the solid-index layer B. Layers C and B were of equal thickness.

**Figure 5:**
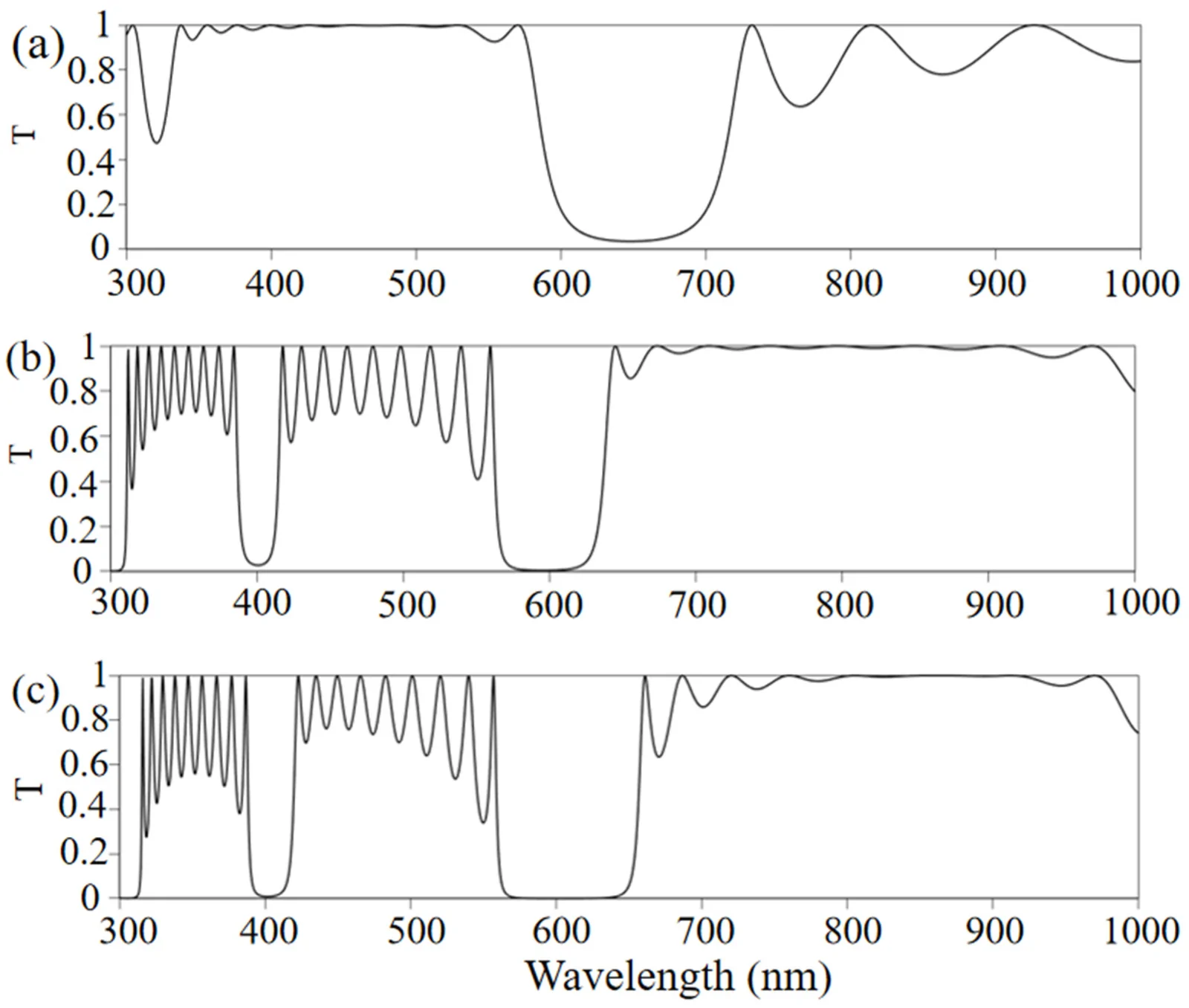
Transmission spectra of three different asymmetric photonic crystal (AC)^6^ with different graded-index films at normal incidence. The graded-index in layer C is from (**a**) 1.60 to 1.00, (**b**) 2.65 to 1.55, and (**c**) 3.30 to 1.10.

To investigate the influence of the gradient refractive-index profiles, three different configurations of the graded-index layer C were considered, namely, linear decreases in the refractive index from 1.60 to 1.00, 2.65 to 1.55, and 3.30 to 1.10. The mean refractive indices of these three graded-index profiles in the asymmetric photonic crystal (AC)^6^ were smaller than, equal to, and larger than the solid refractive index of layer B in the symmetric photonic crystal shown in Figure 1, where d_A_ = 145 nm and d_C_ = 194 nm. The continuous spectral wavelength range where the transmittance satisfies T ≥ 95% is defined as the perfect transmission passband. The bandwidth of this passband is quantified as the wavelength difference between its long-wavelength cutoff and short-wavelength cutoff. Based on the differential transfer matrix method, their transmission spectra were comparatively analyzed at normal incidence to examine the role of the magnitude of the gradient refractive index in regulating the position and width of the perfect transparent band. As shown in Figure 5, the structure with a graded index from 1.60 to 1.00 exhibits a transparent band from 350 nm to 530 nm; the structure with a gradient index from 2.65 to 1.55 exhibits a broad transparent band from 660 nm to 910 nm; and the structure with a gradient from 3.30 to 1.10 exhibits a transparent band from 680 nm to 930 nm. The oscillations in the broad transparent band intensify and the transparent band redshifts as the mean value of the graded index increases.

A comparative analysis of the structures (A_1/2_BA_1/2_)^6^ and (AB)^6^ revealed that the asymmetric one-dimensional photonic crystal with solid-index films cannot exhibit a broad perfect transparent band, as shown in Figure 3a. The symmetric periodic structure with solid-index films possesses a wide perfect transparent band that is jointly governed by multiple Bragg interference effects. In contrast, the asymmetric structure incorporating a graded-index film employs a linear index gradient to achieve smooth optical impedance matching and gradual phase accumulation through its continuous refractive-index profile, thereby breaking the constraints imposed by conventional symmetric photonic crystals. The graded profile with larger mean values exhibits a pronounced redshift of the passband. As shown in Figure 5a, a broad perfect transparent band is formed around 350–530 nm, which is similar to that observed in the mirror-symmetric photonic crystal (A_1/2_BA_1/2_)^6^ with solid-index films, as shown in Figure 3a. Furthermore, the degree of this redshift can be tuned by adjusting the structural parameters such as the gradient refractive-index amplitude and layer thickness, which allows the passband to be shifted to any desired wavelength region, as shown in Figure 5b,c.

**Figure 6:**
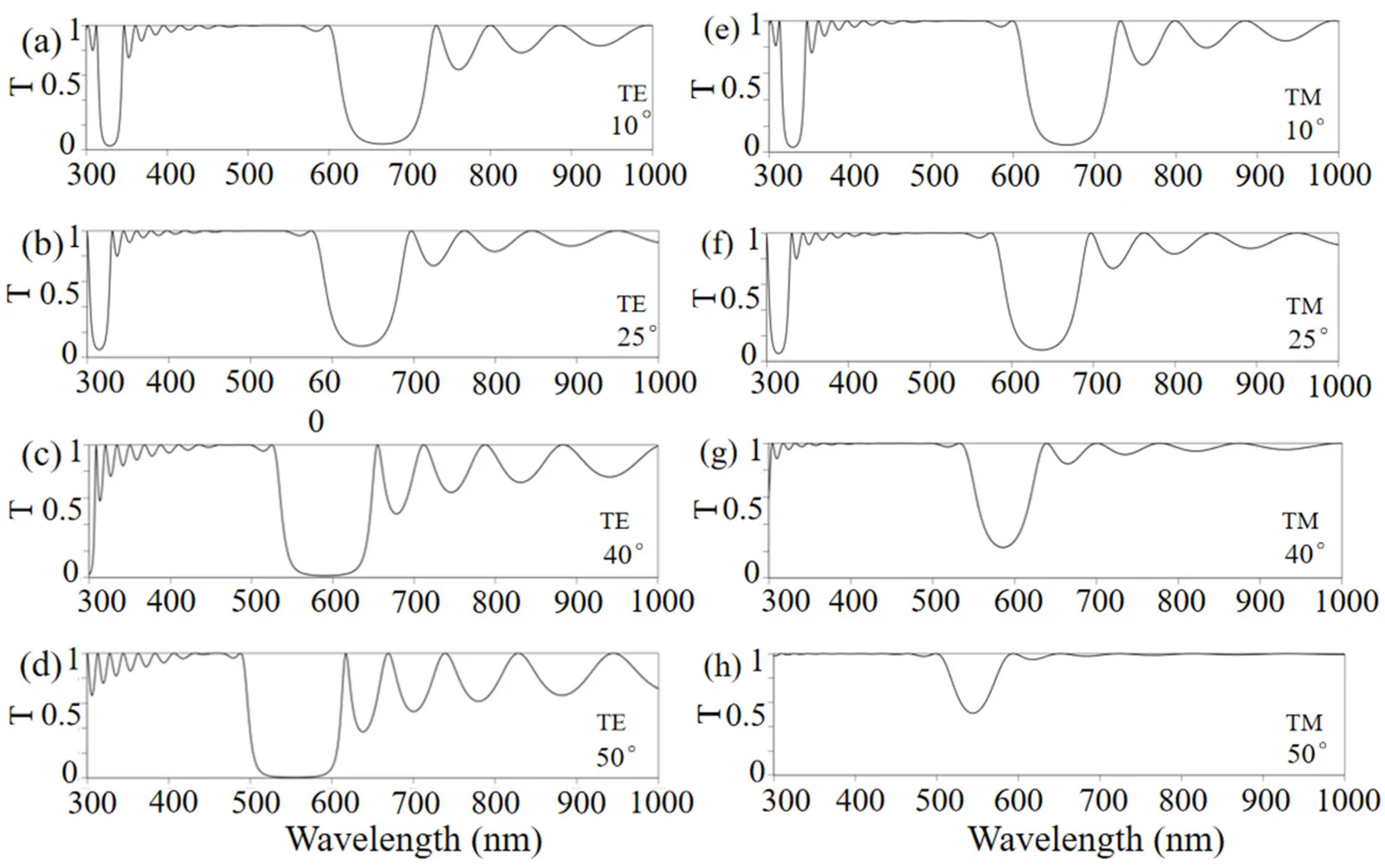
Transmission spectra of the asymmetric one-dimensional photonic crystal (AC)^12^ with graded index from 1.60 to 1.00 at oblique incidence for TE (**a**–**d**) and TM (**e**–**h**) polarizations.

Furthermore, the combined effect of the incident angle and polarization revealed distinct differences. To study the variation trend of the perfect transparent band at two different polarizations, we varied the incident angle while maintaining the period number at *N* = 12. Figure 6 shows the asymmetric photonic crystal with the graded-index profile from 1.60 to 1.00. As shown in Figure 6a–d, a transparent band exists between incident angles of 0° to 50°; however, it exhibits a significant redshift, becomes narrow, and has obvious volatility when the incident angle reaches 50° for TE polarization. Under TM polarization, the same angular variation additionally induces a blue shift in the transparent band; however, the perfect passband remains considerably flatter at a large incident angle, as shown in Figure 6e–h.

**Figure 7:**
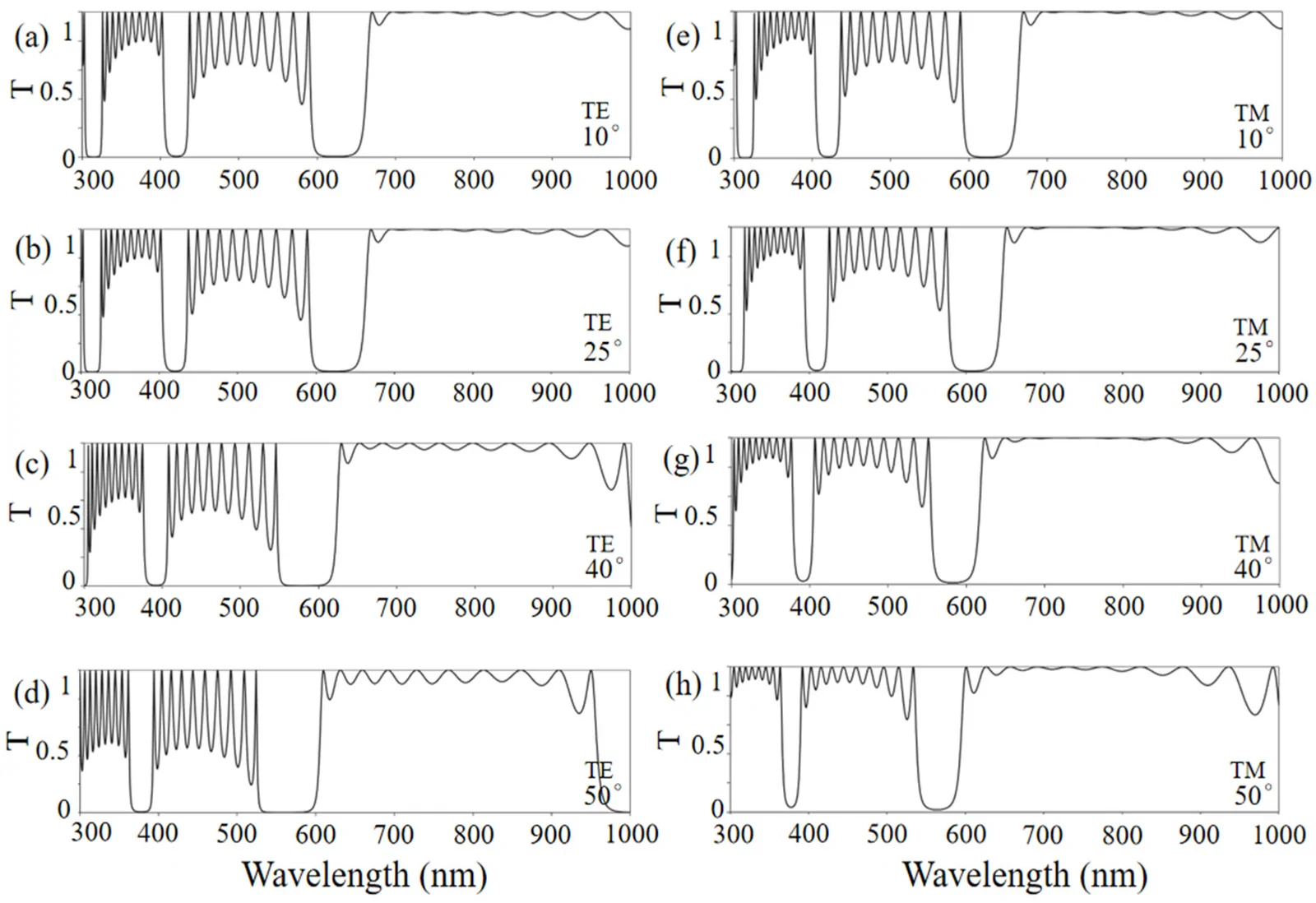
Transmission spectra of the asymmetric one-dimensional photonic crystal (AC)^12^ with graded index from 2.65 to 1.55 at oblique incidence for TE (**a**–**d**) and TM (**e**–**h**) polarizations.

Moreover, we constructed an asymmetric photonic crystal with a graded index that decreased linearly from 2.65 to 1.55, and analyzed the variation trends governing the influence of the oblique incident angle on the broad perfect transparent band. As shown in Figure 7, the passband undergoes a blue shift under both TE and TM polarizations, while the changes in the bandwidth and oscillation magnitude are consistent with the trends observed in Figure 6. The angular stability of TM polarization originates from the distinct angle dependence of effective optical admittance for two polarizations, as shown in Equation (9), where the effective optical admittance for TE polarization decreases with the increase in incident angle, which aggravates impedance mismatch and enhances the reflection; however, the effective optical admittance for TM polarization increases with the incident angle, which nearly maintains impedance matching under oblique incidence. Therefore, the broad perfect transparent band occurring in this asymmetric structure exhibits better angular tolerance for TM polarization. Future studies are required to fabricate experimental samples for validating the simulation results and exploring their potential applications in broadband transparent filters and smart optical sensing.

## 4. Conclusions

In this study, we numerically investigated the broadband perfect transmission properties of one-dimensional asymmetric photonic crystals incorporating a linearly graded-index thin film. The magnitude of the graded refractive-index profile significantly influenced the passband behavior. When the mean value of the graded index increased, the perfect transmission band was redshifted, its bandwidth narrowed, and the transmittance oscillations intensified with increasing incident angles. Moreover, a broad perfect transparent band was realized over a large incident range for both TE and TM polarizations; however, it exhibited greater angular tolerance for TM polarization than for TE polarization. These asymmetric nanostructures containing graded-index films are important for future experiments and applications in optical devices with a perfect broadband transparency.

## Data Availability

The data cannot be made publicly available upon publication because no suitable repository exists for hosting data in this field of study. The data that support the findings of this study are available upon reasonable request from the authors.

## References

[1] Yablonovitch E. (1987). Inhibited spontaneous emission in solid-state physics and electronics. Phys. Rev. Lett..

[2] John S. (1987). Strong localization of photons in certain disordered dielectric superlattices. Phys. Rev. Lett..

[3] Joannopoulos J.D., Villeneuve P.R., Fan S. (1997). Photonic Crystals: Putting a New Twist on Light. Nature.

[4] Butt M.A., Khonina S.N. (2024). Recent Advances in Photonic Crystal and Optical Devices. Crystals.

[5] Hu Y., Tian Z.Q., Ma D.K., Qi C.Z., Yang D.P., Huang S.M. (2024). Smart colloidal photonic crystal sensors. Adv. Colloid Interface Sci..

[6] Lee M., Kim G., Jung Y., Pyun K.R., Lee J., Kim B.-W., Ko S.H. (2023). Photonic structures in radiative cooling. Light Sci. Appl..

[7] Wang Y., Qin H., Mo X., Du G. (2025). Direction-reversible all-optical diode based on a photonic heterostructure. Opt. Lett..

[8] Yu X., Qin H., Li J., Chen H., Li X., Liu F., Wang T., Lu G., Du G. (2025). Anomalous Nonlinear Optical Effects by Intensity-Dependent Phase-Variation Compensation in Photonic Crystals Containing Hyperbolic Metamaterials. Nanomaterials.

[9] Xie M., Huang Y., Qin H., Du G. (2025). Abnormal Angle-Dependent Multi-Channel Filtering in Photonic Crystals Containing Hyperbolic Metamaterials. Nanomaterials.

[10] Southwell W.H. (1983). Gradient-index antireflection coatings. Opt. Lett..

[11] Bulkin P.V., Swart P.L., Lacquet B.M. (1996). Fourier-transform design and electron cyclotron resonance plasma-enhanced deposition of lossy graded-index optical coatings. Appl. Opt..

[12] Smolyaninov I.I. (2021). Gradient-index nanophotonics. J. Opt..

[13] Lyu J., Shen S., Chen L., Zhu Y., Zhuang S. (2023). Frequency selective fingerprint sensor: The terahertz unity platform for broadband chiral enantiomers multiplexed signals and narrowband molecular AIT enhancement. Photonix.

[14] Johnson S.G., Mekis A., Fan S., Joannopoulos J. (2001). Molding the flow of light. Comput. Sci. Eng..

[15] Scalora M., Bloemer M.J., Pethel A.S., Dowling J.P., Bowden C.M., Manka A.S. (1998). Transparent, metallo-dielectric, one-dimensional, photonic band-gap structures. J. Appl. Phys..

[16] Kalozoumis P.A., Morfonios C., Palaiodimopoulos N., Diakonos F.K., Schmelcher P. (2013). Local symmetries and perfect transmission in aperiodic photonic multilayers. Phys. Rev. A.

[17] Du G.Q., Li L.X., Jiang H.T., Li Y.H., Zhang L.W., Zhao J.F., Yang T.L., Wu Z.C., Song S.M., Li Y.H. (2011). Broad flattop transparent photonic band in truncated photonic crystals composed of the symmetric unit cell. Proceedings SPIE 7995, Seventh International Conference on Thin Film Physics and Applications.

[18] Li M., Liu Y., Du L., Li P., Dong Y., Tao L., Li Z., Guo Y., Song K., Zhao X. (2024). Ultrabroadband valley transmission and corner states in valley photonic crystals with dendritic structure. Commun. Phys..

[19] Nava R., Taguena-Martinez J., Del Rio J.A., Naumis G.G. (2009). Perfect light transmission in Fibonacci arrays of dielectric multilayers. J. Phys. Condens. Matter.

[20] Ruiz-Pérez A.D., Reyes-Esqueda J.A. (2021). Generalization of microcavities: Effects of asymmetry in the field localization and transmittance for supported microcavities. Photon. Nanostruct. Fundam. Appl..

[21] Bipin K.S., Vaishali B., Subhashish T., Kaushal K.S., Praveen C.P., Rastogi V. (2021). Photonic band gap consequences in one-dimensional exponential graded index photonic crystals. Optik.

[22] Winn J.N., Fink Y., Fan S., Joannopoulos J.D. (1998). Omnidirectional reflection from a one-dimensional photonic crystal. Opt. Lett..

[23] Fu L., Lin M., Liang Z., Wang Q., Zheng Y., Ouyang Z. (2022). The Transmission Properties of One-Dimensional Photonic Crystals with Gradient Materials. Materials.

[24] Katsidis C.C., Siapkas D.I. (2002). General transfer-matrix method for optical multilayer systems with coherent, partially coherent, and incoherent interference. Appl. Opt..

[25] Suthar B., Bhargava A. (2023). Enhanced optical sensor for waterborne bacteria-based photonic crystal using graded thickness index. Appl. Nanosci..

[26] Kiorpelidis I., Kalozoumis P., Theocharis G., Achilleos V., Diakonos F.K., Pagneux V. (2025). Robustness of perfect transmission resonances to asymmetric perturbation. Phys. Rev. A.

[27] Chau Y.-F.C. (2026). Converging nanophotonics and environmental science: Plasmonic biosensors for smart, distributed monitoring. Environ. Chem. Eng..

[28] Chae S., Lee C., Kim Y., Park Y., Kim Y., Lee S., Kim U.J. (2025). Near-infrared antireflection coating operated with broad incident angles. J. Korean Phys. Soc..

[29] Yeh P. (1988). Optical Waves in Layered Media.

[30] Khorasani S., Mehrany K. (2003). Differential transfer-matrix method for solution of one-dimensional linear nonhomogeneous optical structures. J. Opt. Soc. Am. B.

[31] Hass G. (1975). Physics of Thin Films.

